# An Unusual Complete Response to Immunotherapy in a Locoregional Recurrent Merkel Cell Carcinoma

**DOI:** 10.7759/cureus.77152

**Published:** 2025-01-08

**Authors:** Claudia Correa Roa, Francisco Laxague, Juan Manuel Fernandez Vila

**Affiliations:** 1 Oncological Surgery, Hospital Aleman, Buenos Aires, ARG

**Keywords:** immunotherapy, merkel cell carcinoma, nivolumab, surgical resection

## Abstract

Merkel cell carcinoma (MCC) is a rare cutaneous neuroendocrine carcinoma. Multimodal treatment is associated with improved locoregional control and survival outcomes. We present the case of an 80-year-old male patient who developed MCC on the left arm. After surgical resection with clear margins and a negative sentinel lymph node biopsy (SLNB), the patient remained under follow-up. Nine months later, local recurrence with left axillary lymph node involvement was diagnosed. An oncologic multidisciplinary team recommended neoadjuvant nivolumab. After completing six cycles of immunotherapy, a radical resection of the tumor on the left upper limb and a complete left axillary lymph node dissection were performed. The final histopathological report showed scar fibrosis with no residual cancer and no lymph node metastasis. MCC exhibits immunological sensitivity, and the use of immune checkpoint inhibitors has revolutionized the treatment of advanced and recurrent disease.

## Introduction

Merkel cell carcinoma (MCC) is a rare neuroendocrine skin cancer. Factors contributing to MCC development include age, ultraviolet (UV) radiation exposure, immunosuppression, and Merkel cell polyomavirus infection. Surgical resection is the primary treatment, often supplemented by sentinel lymph node biopsy (SLNB) and adjuvant radiation therapy when indicated [1]. Like other cancers, MCCs can evade the immune system, including the downregulation of major histocompatibility complex I expression on the cell surface, which is crucial for presenting tumor antigens to the adaptive immune system [2]. Remarkably, MCC tumor cells, along with dendritic cells and macrophages in the tumor microenvironment, express PD‐L1 and PD‐L2 ligands. These findings support the use of immune checkpoint inhibitors (ICIs) in treating MCC [3]. This study presents a case of locoregional recurrent MCC treated with neoadjuvant immunotherapy followed by surgical resection.

## Case presentation

**Figure 1:**
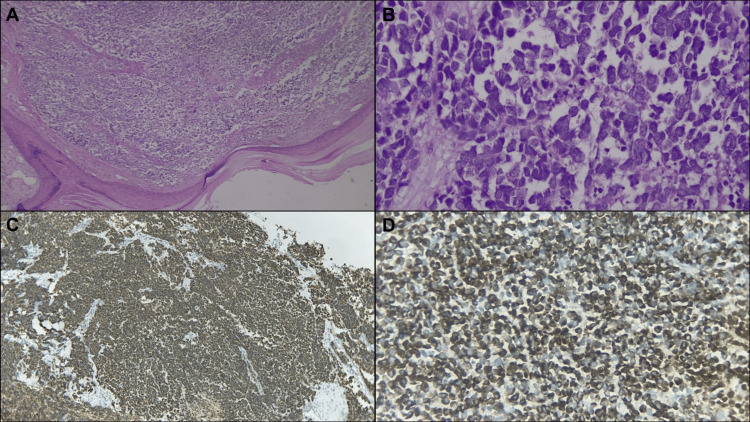
Histological features of Merkel cell carcinoma (A and B) Microscopic features of MCC on hematoxylin-eosin staining (4x and 10x high-power-field (HPF), respectively). (C and D) The tumor showed CK20 paranuclear dot-like staining, along with positive staining for CD56, chromogranin, and synaptophysin (4x and 10x HPF, respectively).

An 80-year-old male patient with no relevant clinical history presented with an ulcerated, bleeding skin lesion on his left arm. Physical examination revealed a 2-cm raised, ulcerated lesion on the inner surface of the arm. A surgical resection was performed, and the histopathologic report confirmed MCC, with a tumor size of 1.5 cm and a thickness of 70 mm, exhibiting a nodular growth pattern and clear margins. Immunohistochemistry showed CK20 paranuclear dot-like staining and neuroendocrine features, including chromogranin and synaptophysin (Figure 1).

**Figure 2:**
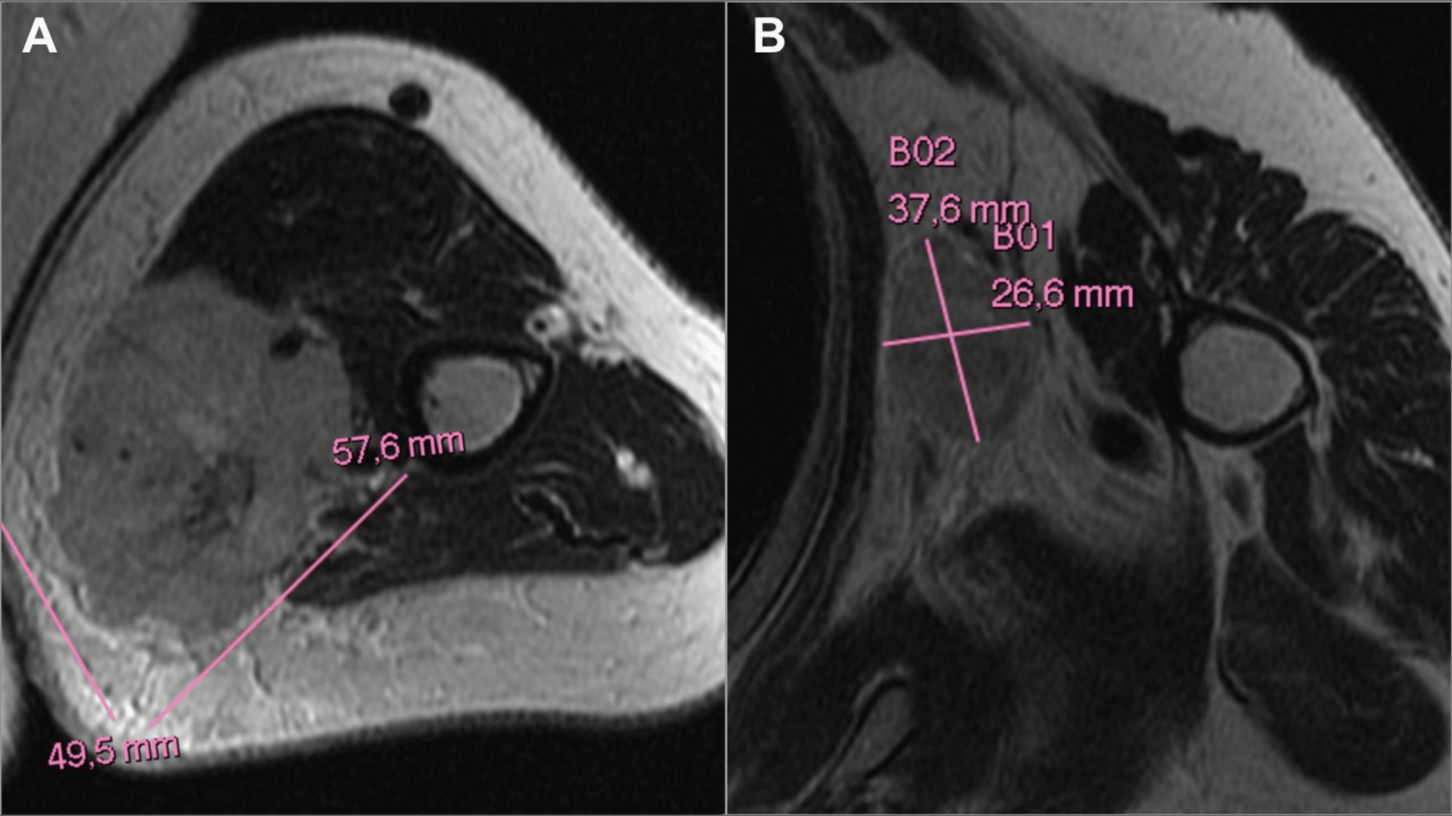
Initial magnetic resonance imaging MRI images show the left arm tumor and its relationship with the vasculonervous structures of the humeral bundle and axillary lymphadenopathy, respectively. (A) T2 sequence. (B) T1 sequence.

Subsequently, a radical resection and an SLNB were conducted. The histopathologic report confirmed the absence of residual neoplastic cells and metastasis. After a disease-free period of nine months, the patient developed a 4-cm mass on the left arm. Physical examination revealed a 5-cm hard, fixed mass, accompanied by painful left axillary lymphadenopathy. A fine-needle aspiration (FNA) biopsy of the mass was performed, confirming infiltration by MCC. A positron emission tomography-computed tomography (PET-CT) scan showed increased uptake and metabolic activity in the lesion on the left arm, with involvement of the left brachial and axillary lymph nodes. A magnetic resonance imaging (MRI) revealed a 5.7 x 4.9 cm lesion on the inner side of the left arm involving the vasculonervous structures of the humeral bundle and axillary lymphadenopathy measuring 3.7 x 2.6 cm (Figure 2).

**Figure 3:**
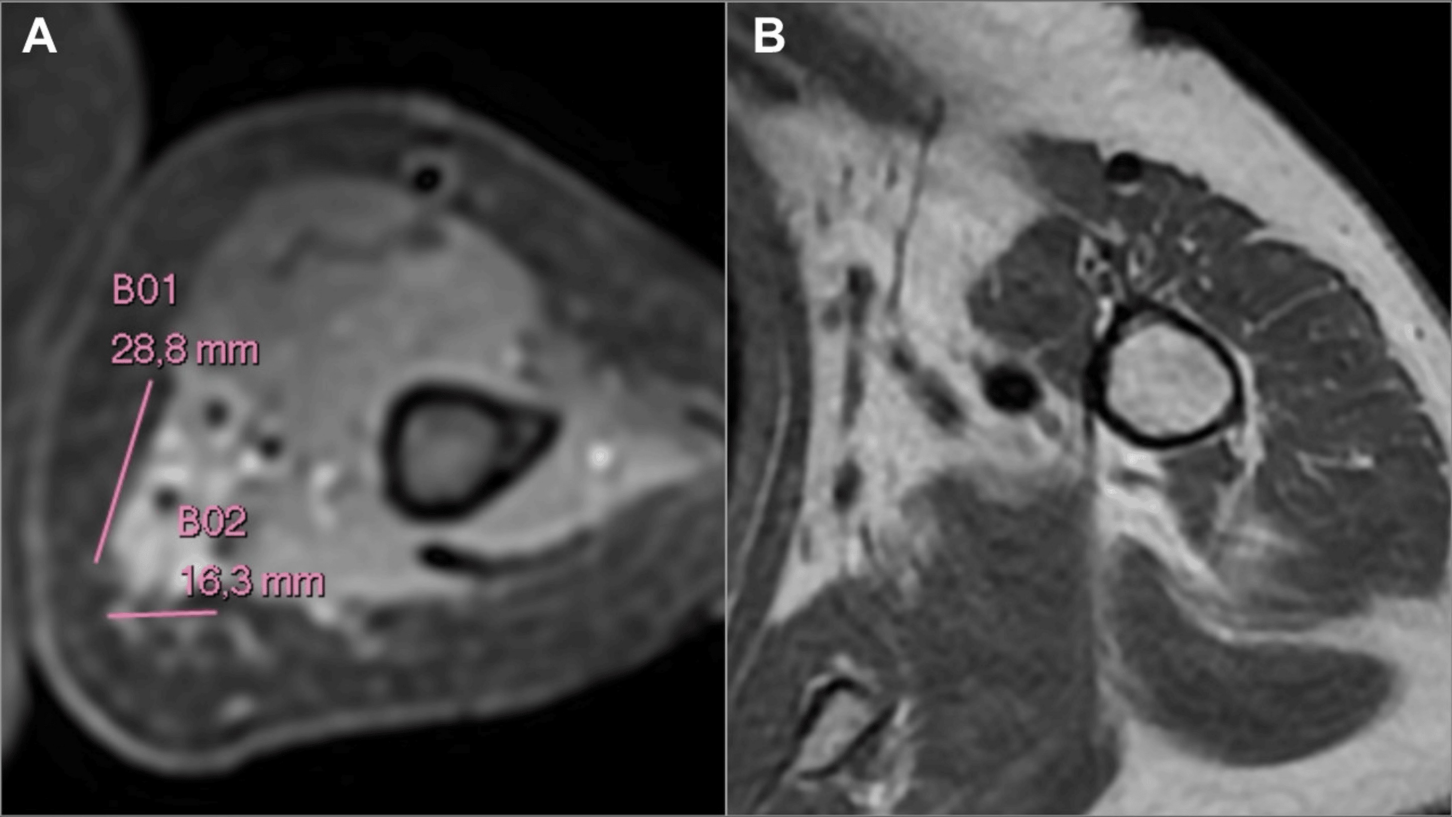
After-treatment MRI MRI after completing six cycles of nivolumab shows a reduction in the size of the lesion in the left arm and no signs of axillary lymphadenopathy. (A) T2 STIR sequence. (D) T1 sequence.

Due to recurrence with locally advanced involvement, the case was discussed in a multidisciplinary oncologic committee, and neoadjuvant immunotherapy with nivolumab was recommended, with the patient completing six cycles. A follow-up MRI was performed, revealing a persistent lesion on the inner side of the left arm, measuring 2.8 x 1.6 cm, in contact with the vasculonervous structures of the humeral bundle, with no evidence of the previously noted axillary lymphadenopathy (Figure 3).

A radical resection of the tumor was performed, involving the basilic vein and the internal cutaneous brachial nerve, which were affected by the tumor, while preserving the brachial nerve and humeral vessels. Additionally, a complete left axillary lymph node dissection was performed. The histopathological report showed scar fibrosis with no residual neoplastic cells and no lymph node compromise. After a multidisciplinary committee discussion, adjuvant radiotherapy was recommended (5000 cGy intensity-modulated radiation therapy (IMRT)) for the left arm and the ipsilateral axilla. After 18 months of follow-up, the patient remained disease-free with good arm function.

## Discussion

This study presents a case of locoregional recurrent MCC treated with immunotherapy and discusses emerging data on immunotherapy for advanced MCC. To our knowledge, this is the first report of a complete response to immunotherapy in a locoregional recurrent MCC.

MCC presents on sun-exposed skin as an asymptomatic ulcerated nodular lesion, often leading to misdiagnosis [1]. The characteristic histology of MCC shows small, round cells with high mitotic activity. Immunohistochemistry is classically positive for CK20, neuroendocrine markers (such as synaptophysin and chromogranin), and negative for CK7 and TTF-1 [1,4-6]. Patients diagnosed with biopsy-confirmed MCC may undergo further evaluation, including imaging studies, as clinically indicated. Recommended imaging includes MRI, CT scan, or whole-body FDG PET-CT [5,7]. MCCs present an aggressive biology and are associated with frequent recurrences, metastasis, and a high mortality rate. Metastatic sites include lymph nodes, nonregional skin sites, bones, and subcutaneous tissue [1,6-8].

Treatment depends on the histopathological interpretation and staging of the primary lesion. Surgery is the primary treatment, with the main objective being complete tumor excision with wide margins, depending on the tumor’s thickness [7,9]. Surgical resection is also necessary for staging the primary lesion and regional disease [1].

Most patients present with nodal or distant spread at presentation, making SLNB valuable for identifying subclinical nodal metastases. A positive SLNB result warrants complete dissection of the affected lymph node basin, which is also indicated in clinically positive disease. In unresectable cases, radiation therapy becomes essential, targeting the lymph node tumor drainage basin [1]. MCC demonstrates high radiosensitivity, so postoperative radiotherapy is recommended to reduce the risk of local recurrence [7-9]. Adjuvant radiotherapy after lymph node dissection is reserved for patients with multiple involved nodes and/or extracapsular extension. If SLNB is negative, observation of the nodal basin is appropriate. For patients with distant metastases, individualized approaches are needed, with systemic therapy and radiotherapy emerging as primary treatment options [7,8].

While MCC is sensitive to chemotherapy, the high response rates are often short-lived, with rapid recurrence [2,9]. Chemotherapy also carries a significant risk of toxicity, especially in older patients. As a result, there is a growing interest in using ICIs, such as avelumab, pembrolizumab, and nivolumab, in the adjuvant setting [8]. ICIs produce rapid responses comparable to those seen with frontline chemotherapy but tend to be more durable. These responses are associated with a better quality of life and may significantly improve overall survival, offering the potential for a cure in what is typically a terminal condition. Treatment-naïve patients generally have higher response rates to ICIs than those who have undergone prior chemotherapy, likely due to the immunosuppressive effects of chemotherapy [2,9]. Therefore, PD-1-/PD-L1-based immunotherapy should be considered the new standard of care for the initial treatment of patients with advanced or recurrent MCC [8,9]. 

Topalian et al. [10] reported on the CheckMate 358 phase I/II trial, in which 39 patients with resectable stage IIA-IV MCC received one or more doses of nivolumab. Of these, 36 patients underwent surgery, while three did not due to disease progression or adverse events. Among the surgically treated patients, a 47.2% pathologic complete response rate was achieved. Additionally, in 54.5% of the surgically treated patients who underwent radiological evaluation, more than a 30% reduction in tumor size was observed. The median follow-up for this cohort was 20.3 months, and none of the patients who achieved a complete response experienced tumor recurrence during the observation period. This study demonstrated a complete pathological response after immunotherapy in a locoregional advanced MCC.

Numerous promising immune-based treatment approaches are currently under investigation, and the recent progress in the development of immunotherapy agents holds the promise of offering effective treatment alternatives for patients with advanced or recurrent MCC in the near future.

## Conclusions

MCC is a rare neuroendocrine skin cancer. In addition to surgery and radio-/chemotherapy, immunotherapy provides a promising treatment alternative for patients with primary and recurrent MCC. Future studies with larger patient cohorts are needed to provide evidence supporting this treatment approach.
